# On Unsoundness of Mind in Its Legal and Medical Considerations

**Published:** 1891-03

**Authors:** 


*On Unsoundness of Mind in its Legal and Medical Considerations.* By J. W. HUME WILLIAMS. London: WM. CLOWES & SONS, LIMITED. 1890.

This book is a collection of essays on insanity from various aspects, primarily written at different times, and now brought together by the author, a barrister. From internal evidence, it would appear that these essays were written in their original, if not present, form a considerable number of years ago, in which case we must congratulate Mr. Hume Williams upon the very humane views which he takes upon such questions as moral and impulsive insanity. This is professedly not a text-book, hardly calling therefore for detailed criticism in these pages; so we will not do more than compliment the author on his display of legal and general learning, and thank him for the generous manner in which he speaks of medicine and the difficulties which surround the investigation of mental phenomena. With a distinct metaphysical bias, the author has given us an interesting and suggestive book, well worth reading by those engaged in the study of psychology.

